# Vitamin D-Related Genes, Blood Vitamin D Levels and Colorectal Cancer Risk in Western European Populations

**DOI:** 10.3390/nu11081954

**Published:** 2019-08-20

**Authors:** Veronika Fedirko, Hannah B. Mandle, Wanzhe Zhu, David J. Hughes, Afshan Siddiq, Pietro Ferrari, Isabelle Romieu, Elio Riboli, Bas Bueno-de-Mesquita, Fränzel J.B. van Duijnhoven, Peter D. Siersema, Anne Tjønneland, Anja Olsen, Vittorio Perduca, Franck Carbonnel, Marie-Christine Boutron-Ruault, Tilman Kühn, Theron Johnson, Aleksandrova Krasimira, Antonia Trichopoulou, Periklis Makrythanasis, Dimitris Thanos, Salvatore Panico, Vittorio Krogh, Carlotta Sacerdote, Guri Skeie, Elisabete Weiderpass, Sandra Colorado-Yohar, Núria Sala, Aurelio Barricarte, Maria-Jose Sanchez, Ramón Quirós, Pilar Amiano, Björn Gylling, Sophia Harlid, Aurora Perez-Cornago, Alicia K. Heath, Konstantinos K. Tsilidis, Dagfinn Aune, Heinz Freisling, Neil Murphy, Marc J. Gunter, Mazda Jenab

**Affiliations:** 1 Rollins School of Public Health, Emory University, Atlanta, GA 30322, USA; 2 Winship Cancer Institute, Emory University, Atlanta, GA 30322, USA; 3 Cancer Biology and Therapeutics Group (CBT), Conway Institute, School of Biomolecular and Biomedical Science (SBBS), University College Dublin, Dublin, Ireland; 4 Genomics England, London EC1M 6BQ, UK; 5 Imperial College London, London SW7 2AZ, UK; 6 Section of Nutrition and Metabolism, International Agency for Research on Cancer (IARC-WHO), Lyon 69372, France; 7 Department of Epidemiology and Biostatistics, School of Public Health, Imperial College London, London SW7 2AZ, UK; 8 Division of Human Nutrition & Health, Wageningen University & Research, 6700 AA Wageningen, The Netherlands; 9 Department of Gastroenterology and Hepatology, Radboud University Medical Center, 6525 GA Nijmegen, The Netherlands; 10 Danish Cancer Society Research Center, 2100 Copenhagen, Denmark; 11 Laboratoire de Mathématiques Appliquées MAP5, Université Paris Descartes, 75006 Paris, France; 12 CESP, Fac. de médecine—Univ. Paris-Sud, Fac. de médecine—UVSQ, INSERM, Université Paris-Saclay, F-94805 Villejuif, France; 13 Gustave Roussy, F-94805 Villejuif, France; 14 Department of Gastroenterology, Bicêtre University Hospital, Assistance Publique des Hôpitaux de Paris, 94270 Le Kremlin Bicêtre, France; 15 Division of Cancer Epidemiology, German Cancer Research Center (DKFZ), 69120 Heidelberg, Germany; 16 Nutrition, Immunity and Metabolism, Department of Epidemiology, German Institute for Human Nutrition Potsdam-Rehbrücke, Arthur-Scheunert Allee, 14558 Nuthetal, Germany; 17 Hellenic Health Foundation, 115 27 Athens, Greece; 18 Biomedical Research Foundation of the Academy of Athens, 115 27 Athens, Greece; 19 Dipartimento Di Medicina Clinica E Chirurgia, Federico Ii University, 80138 Naples, Italy; 20 Epidemiology and Prevention Unit, Fondazione IRCCS Istituto Nazionale dei Tumori, Via Venezian, 20133 Milano, Italy; 21 Unit of Cancer Epidemiology, Città della Salute e della Scienza University-Hospital and Center for Cancer Prevention (CPO), 10126 Turin, Italy; 22 Department of Community Medicine, Faculty of Health Sciences, University of Tromsø, The Arctic University of Norway, 9019 Tromsø, Norway; 23 Department of Research, Cancer Registry of Norway, Institute of Population-Based Cancer Research, N-0304 Oslo, Norway; 24 Department of Medical Epidemiology and Biostatistics, Karolinska Institut, SE-171 77 Stockholm, Sweden; 25 Genetic Epidemiology Group, Folkhälsan Research Center and Faculty of Medicine, Helsinki University, Helsinki 00014, Finland; 26 International Agency for Research on Cancer (IARC-WHO), Lyon 69372, France; 27 Department of Epidemiology, Murcia Regional Health Council, IMIB-Arrixaca, Murcia 30008, Spain; 28 CIBER Epidemiology and Public Healh (CIBERESP), Madrid 28029, Spain; 29 Research Group on Demography and Health, National Faculty of Public Health, University of Antioquia, Cl. 67 ##53-108 Medellín, Colombia; 30 Unit of Nutrition and Cancer, Cancer Epidemiology Research Program, and Translational Research Laboratory, Catalan Institute of Oncology (ICO)-IDIBELL, 08908 Barcelona, Spain; 31 Navarra Public Health Institute, Pamplona 31008, Spain; 32 Escuela Andaluza de Salud Pública, Instituto de Investigación Biosanitaria (ibs.GRANADA), Granada 18012, Spain; 33 Public Health Directorate, Asturias 33006, Spain; 34 Public Health Division of Gipuzkoa, BioDonostia Research Institute, San Sebastian 20014, Spain; 35 Department of Medical Biosciences, Pathology, Umeå University, 901 87 Umeå, Sweden; 36 Department of Radiation Sciences, Oncology, Umeå University, 901 87 Umeå, Sweden; 37 Cancer Epidemiology Unit, Nuffield Department of Population Health, University of Oxford, Oxford OX3 7LF, UK; 38 Department of Hygiene and Epidemiology, University of Ioannina School of Medicine, Ioannina 45110, Greece; 39 Department of Nutrition, Bjørknes University College, 0456 Oslo, Norway; 40 Department of Endocrinology, Morbid Obesity and Preventive Medicine, Oslo University Hospital, 0372 Oslo, Norway

**Keywords:** single nucleotide polymorphism (SNP), vitamin D, colorectal neoplasms, incidence

## Abstract

Higher circulating 25-hydroxyvitamin D levels (25(OH)D) have been found to be associated with lower risk for colorectal cancer (CRC) in prospective studies. Whether this association is modified by genetic variation in genes related to vitamin D metabolism and action has not been well studied in humans. We investigated 1307 functional and tagging single-nucleotide polymorphisms (SNPs; individually, and by gene/pathway) in 86 vitamin D-related genes in 1420 incident CRC cases matched to controls from the European Prospective Investigation into Cancer and Nutrition (EPIC) cohort. We also evaluated the association between these SNPs and circulating 25(OH)D in a subset of controls. We confirmed previously reported CRC risk associations between SNPs in the *VDR*, *GC*, and *CYP27B1* genes. We also identified additional associations with 25(OH)D, as well as CRC risk, and several potentially novel SNPs in genes related to vitamin D transport and action (*LRP2, CUBN, NCOA7*, and *HDAC9*). However, none of these SNPs were statistically significant after Benjamini–Hochberg (BH) multiple testing correction. When assessed by a priori defined functional pathways, tumor growth factor β (TGFβ) signaling was associated with CRC risk (*P* ≤ 0.001), with most statistically significant genes being *SMAD7 (P_BH_* = 0.008) and *SMAD3 (P_BH_* = 0.008), and 18 SNPs in the vitamin D receptor (VDR) binding sites (*P* = 0.036). The 25(OH)D-gene pathway analysis suggested that genetic variants in the genes related to VDR complex formation and transcriptional activity are associated with CRC depending on 25(OH)D levels (interaction *P* = 0.041). Additional studies in large populations and consortia, especially with measured circulating 25(OH)D, are needed to confirm our findings.

## 1. Introduction

Colorectal cancer (CRC) is the second most common cancer in men and women combined, with approximately 1.4 million new cases diagnosed in 2012 worldwide [1]. There is compelling observational evidence that low circulating vitamin D concentrations are associated with increased risk of incident CRC [2,3]. However, other human evidence is less convincing. A few Mendelian randomization (MR) studies did not support an association between vitamin D genetic score and CRC risk, but the genetic contribution to 25(OH)D is relatively small (7.5% as estimated based on genome-wide association studies (GWAS) on common SNPs [4]), possibly explaining the null findings [5,6]. Also, the relatively few randomized clinical trials (RCTs) of vitamin D supplementation and colorectal neoplasms have not shown statistically significant effects, but sample size, duration and timing of supplementation, issues with compliance and choice of study population, and the limited range of vitamin D exposures assessed may have contributed to the null results [7,8,9]. Finally, the benefits from vitamin D supplementation for the prevention of colorectal neoplasms may vary according to genetic variation in the vitamin D-related genes (e.g., vitamin D receptor (*VDR*) [10]). 

Anti-neoplastic effects of vitamin D on colorectal tissue are also supported by the fact that the normal colorectal epithelium expresses the vitamin D receptor (VDR) and vitamin D metabolizing enzymes (CYP27B1 and CYP24A1) and, therefore, can locally produce and degrade the active form of vitamin D, 1,25-dihydroxyvitamin D (1,25(OH)_2_D), from 25-hydroxyvitamin D (25(OH)D) [11,12,13]. In the colorectum, the active metabolite of vitamin D, 1,25(OH)_2_D, exerts its anti-neoplastic effects by genomic (mediated by the VDR) and non-genomic mechanisms [14], including the regulation of over 200 vitamin D-responsive genes and rapid activation of intracellular signaling pathways, resulting in modulation of the cell cycle, bile acid degradation, immune response, growth factor signaling, and anti-inflammation [15]. 

Observational and RCT data suggest a potential vitamin D-colorectal neoplasms risk association is modified by polymorphisms in the vitamin D receptor (VDR) [10,16,17] and the vitamin D-binding protein gene (GC) [18]; however, only a few single nucleotide polymorphisms (SNPs) and a limited number of related pathways were considered. Novel evidence highlights a wide array of VDR binding sites across the human genome [19], and multiple pathways related to vitamin D effects [20]. Thus, it is plausible that the vitamin D–CRC risk association may be modulated by variation in a broad array of genes related to vitamin D metabolism (e.g. absorption, endogenous synthesis, transport, activation, and deactivation) and action (including transcriptional activity/post-transcriptional effects). All of these genes are polymorphic, but no studies to date have comprehensively investigated their individual and collective associations with CRC risk or circulating vitamin D levels. In consideration of these points, we investigated whether variation in genes related to vitamin D metabolism and transcriptional activity is related to circulating blood vitamin D levels, and whether genetic variation at the SNP, pathway and gene level, alone and in combination with circulating vitamin D levels, is associated with CRC risk in a large Western European prospective cohort study.

## 2. Materials and Methods

### 2.1. Study Population 

We used a case-control design nested within the European Prospective Investigation into Cancer and Nutrition (EPIC) cohort, a large prospective study with over 520,000 men and women aged 35–70 years enrolled from 23 centers in 10 Western European countries (Denmark, France, Greece, Germany, Italy, the Netherlands, Norway, Spain, Sweden, and United Kingdom). The methods of the EPIC study have been described in detail elsewhere [21,22]. Individuals who were eligible for the study were selected from the general population of a specific geographical area, town, or province. Exceptions included the French sub-cohort, which is based on members of the health insurance system or state-school employees, and the Utrecht (Netherlands) sub-cohort, which is based on women who underwent screening for breast cancer. Between 1992 and 1998, standardized lifestyle and personal history information, anthropometrics, and blood samples were collected from most participants at recruitment. Diet over the previous 1 year was measured at baseline by validated country-specific dietary questionnaires developed to ensure high compliance and better measures of local dietary habits [21]. Blood samples were stored at the International Agency for Research on Cancer (Lyon, France; −196 °C, in liquid nitrogen) for all countries except Denmark (−150 °C, in nitrogen vapor) and Sweden (in −80 °C freezers). The EPIC study was approved by the Ethical Review Board of the International Agency for Research on Cancer (IARC) and the Institutional Review Board of each participating EPIC center. Written consent was obtained from all EPIC participants at enrolment into the study. 

### 2.2. Cancer Incidence and Vital Status Follow-Up

Cancer incidence was determined through record linkages with regional cancer registries (Denmark/Italy/the Netherlands/Norway/Spain/Sweden/United Kingdom; complete up to December 2006) or via a combination of methods, including the use of health insurance records, contacts with cancer and pathology registries, and active follow-up through study subjects and their next-of-kin (France/Germany/Naples/Greece; complete up to June 2010).

Vital status follow-up (98.5% complete) was collected by record linkage with regional and/or national mortality registries in all countries except France, Germany, and Greece, where data are collected through an active follow-up. Censoring dates for complete follow-up were between June 2005 and June 2009 in Denmark, the Netherlands, Spain, the United Kingdom, Sweden, Norway, and Italy. In Germany, Greece, and France follow-up was based on a combination of methods, including health insurance records, cancer and pathology registries, and active follow-up through study subjects and their next-of-kin. In these centers, the end of follow-up was defined as the last known date of contact, or the date of death whichever came first. The last update of endpoint information occurred between December 2007 and December 2009. 

### 2.3. Nested Case-Control Design and Participant Selection

#### 2.3.1. Case Ascertainment and Selection

CRC cases were selected among participants who developed colon (C18.0–C18.7, according to the ICD–10), rectum (C19–C20), and overlapping/unspecified origin tumors (C18.8 and C18.9). Cancers of the anus were excluded. CRC is defined as the combination of the colon and rectal cancers. 

A total of 1420 first-time previously cancer-free colorectal cancer cases (colon cancer = 900; rectal cancer = 520) were identified. Cases were not selected from Norway (blood samples only recently collected; few colorectal cancers diagnosed after blood donation) and the Malmö center of Sweden. The number of cases for gene-environment analyses was 1176 because of missing, previously collected 25(OH)D measurements [23] (France = 6, Italy = 49, Spain = 30, UK = 27, The Netherlands = 8, Greece = 18, Germany = 21, and Sweden = 16).

#### 2.3.2. Control Selection

Controls were selected (1:1) by incidence density sampling from all cohort members alive and not having a reported cancer at the time of diagnosis of the cases and were matched by age (±6 months at recruitment), sex, study center, time of the day at blood collection, and fasting status at the time of blood collection (less than three hours, three to six hours, and more than six hours). Women were further matched by menopausal status (pre-/post-/peri-menopausal, and unknown) and for pre-menopausal women, phase of menstrual cycle at time of blood collection and usage of postmenopausal hormone therapy at time of blood collection (yes/no, regardless of menopausal status). The additional matching criteria for women were required for other studies that were being carried out using the same matched case-control sets. One control sample failed the genotyping and was not included in the analysis, resulting in a total of 1419 controls. The number of controls for analyses involving 25(OH)D was 764 because of missing or unobtainable, previously collected 25(OH)D measurements [23] (France = 18, Italy = 69, Spain = 48, UK = 62, The Netherlands = 41, Greece = 23, Germany = 60, Sweden = 49, and Denmark = 328). 

#### 2.3.3. Blood 25-(OH)-Vitamin D Assessment

We previously measured blood concentrations of 25(OH)D using a commercially available enzyme immunoassay kit (OCTEIA 25-(OH)D Kit, Immuno Diagnostic Systems, Boldon, UK) at the Laboratory for Health Protection Research, National Institute for Public Health and the Environment, the Netherlands [23]. The kit is specific for 100% of 25-(OH)-vitamin D_3_ form and 75% of 25-(OH)-vitamin D_2_ form. The inter-assay coefficient of variation as determined with two kit control samples was minimal (5.9% at the level of 20.3 nmol/L and 5.4% at the level of 77.4 nmol/L). No significant between-day drift, time shifts, or other trends were observed and the percentage of variance attributable to batch-to-batch differences was 4.5%. For all analyses, laboratory technicians were blinded to the case-control status of the samples.

#### 2.3.4. SNP Selection, Genotyping, and Quality Control

Genomic DNA was extracted from whole blood samples using conventional methods. We used the custom GoldenGate Universal-32 3072-plex assay kit (Illumina, CA, USA) to genotype 1716 genetic variants within the genes known and proposed to be involved in (1) vitamin D metabolism (*DHCR7, GC, CYP3A4, CYP2R1, CYP24A1, CUBN*, and *LRP2*), (2) mineral homeostasis and endocrine regulations of 1,25(OH)_2_D synthesis (*CASR, PTH, TRPV5*, and *TRPV6*), (3) vitamin D genomic effects (*VDR, RXRA, RXRB*, and *RXRG*), (4) formation of the *VDR* complex (co-activators and co-regulators *ACTL6A, ARID1A, BAZ1B, CARM1, CHAF1A, CREBBP, EP300, HDAC9, MED1, NCOA1, NCOA2, NCOA3, NCOA7, NCOR1, NCOR2, PCAF/KAT2B, PRMT1, SMARCA2, SMARCA4, SMARCC1, SMARCD1, SMARCE1, SNW1, SUPT16H, TOP2B*, and *TSC2*), and (5) vitamin D post-transcriptional response (tumor growth factor β (TGFβ)-signaling, inflammation, oxidative stress, insulin growth factor (IGF) signaling, cell cycle, and *VDR* binding sites; please see Supplementary Table S1 for a complete list of genes and SNPs). The custom GoldenGate assay was designed using the Illumina online Assay Design Tool in May 2012. SNP genotype dataset for CEU population (Utah residents with Northern and Western European ancestry; HapMap Data Rel 28 Phase II + III, August 10, on NCBI B36 assembly, dbSNP b126) were loaded in the Haploview program (Broad Institute, MIT and Harvard, Cambridge, MA, USA) and SNPs with minor allele frequencies (MAFs) greater than 5% and the r^2^ linkage disequilibrium (LD) statistic of 0.8 were selected as tagging SNPs (tagSNPs). Additionally, we searched published literature for previously reported functional and regulatory SNPs in the genes of interest and included them in genotyping irrespective of MAFs or r^2^ with other SNPs. Genotyping was performed by the Genetics Laboratory at Imperial College London. After excluding 409 SNPs [247 (14.4%) that failed genotyping, 54 (3.1%) that failed to satisfy the Hardy-Weinberg criterion (Supplementary Table S1), 98 (5.7%) missing in more than 20% of genotyped samples, and 10 (0.6%) that were monomorphic], a total of 1307 SNPs were included in the analysis. All genotyping underwent standard quality control including concordance checks for blinded duplicates and examination of sample and SNP call rates. The lowest reproducibility frequency across 62 replicate samples was 0.98. The call rate was 95% for all samples and 95% for all SNPs.

#### 2.3.5. Statistical Analysis

The season adjustment of 25(OH)D was carried out by the week of blood draw using the sine curve method [24]. The associations between season-adjusted 25(OH)D concentrations and genetic variants (coded as 0, 1, 2 corresponding to the number of minor alleles) were assessed among controls using linear regression models adjusted for age, sex, and center. Further adjustment for BMI, smoking status, and physical activity did not change the results substantially. We used unconditional logistic regression analysis to assess the association of individual SNPs with CRC risk, adjusting for age (continuous), sex, and study center. Results were similar when we used conditional logistic regression on 1331 complete case-matched sets. We assumed a log-additive genetic model, but also tested dominant and recessive models as the underlying genetic model for these SNPs is unknown. Further adjustment for body mass index (BMI; continuous), smoking status (never, former, current smokers, missing), physical activity (active, moderately active, moderately inactive, and inactive), alcohol intake (continuous), hormone therapy, and menopausal status did not substantially change the results, and thus these variables were not included in the final statistical model. Subgroup analyses were conducted by sex and tumor location (colon vs. rectum).

To examine the associations between genes (a combination of SNPs) and genetic pathways (a combination of genes) and CRC risk, we used the Adaptive Rank Truncated Product (ARTP) method [25] as implemented in the first step (no interaction) of the R package PIGE (http://cran.r-project.org/web/packages/PIGE/index.html). This method can combine associations of SNPs in each gene (or from the genes in a pathway) to provide a *P*-value at the gene or pathway level, respectively. Genetic markers in high LD (r^2^ ≥ 0.8) were excluded using the AdaJoint R package (https://cran.r-project.org/web/packages/ARTP2). To investigate the multiplicative interaction between the genes and genetic pathways with 25(OH)D on CRC risk, we used the modified ARTP method as implemented in the R package PIGE. The *P*-values at the SNP and the gene levels were corrected for multiple testing for the number of SNPs and for the number of genes, respectively, using the false discovery rate (Benjamini–Hochberg or BH) method [26]. Furthermore, we used traditional methods to assess potential interactions between SNPs and 25(OH)D stratifying by categories of 25(OH)D concentrations and assuming a log-additive model for genetic markers. Also, we assessed the association of 25(OH)D (per 24.96 nmoL = 10 ng/mL) with CRC risk by genotype. 

All statistical tests were two-sided with *P*-values < 0.05 considered statistically significant (SAS software, version 9.2; SAS Institute, Cary/NC; R, R Foundation for Statistical Computing, Vienna/Austria).

## 3. Results

### 3.1. Baseline Characteristics of Cases and Controls

Selected baseline characteristics of the CRC cases and matched controls are shown in Table 1. The mean age at blood donation of cases and controls was 58 years. On average, CRC cases had 4 years between blood donation and the time of diagnosis. The dataset included 520 rectal cancer cases and 900 colon cancer cases.

### 3.2. SNPs in the Genes Related to Vitamin D Metabolism/Transcriptional Activity and 25(OH)D

Thirty-seven SNPs in the genes related to vitamin D metabolism, formation of the *VDR* complex, and *VDR* transcriptional activity were associated with season-adjusted 25(OH)D concentrations with unadjusted *P* ≤ 0.05 among controls (Supplementary Table S2). The top 10 SNPs are shown in Table 2. Of the 37, 17 SNPs were in the genes involved in vitamin D metabolism, and 20 SNPs in the genes involved in vitamin D transcriptional activity. None of these SNPs were statistically significantly associated with 25(OH)D after BH correction. The associations of all SNPs with 25(OH)D among controls only are shown in Supplementary Table S3A, and among cases and controls combined in Supplementary Table S3B.

### 3.3. SNPs in the Genes Related to Vitamin D Metabolism/Function and CRC Risk

We examined the associations between SNPs in the genes involved in vitamin D metabolism (genes = 9, SNPs = 274), mineral homeostasis and endocrine regulation of 1,25(OH)_2_D synthesis (genes = 5, SNPs = 58), vitamin D genomic effects including the *VDR* complex co-activators and co-regulators (genes = 30, SNPs = 538), and two SNPs in the intergenic regions previously associated with circulating 25(OH)D [27] and CRC risk (Supplementary Table S4). In Table 3, we show the top fifteen statistically significant SNPs associated with CRC risk defined by *P_unadjusted_* < 0.01. However, after BH correction, none of the associations remained statistically significant (all *P_BH_* > 0.2). The results did not differ by tumor location (Table 3 and Supplementary Table S4) or sex (Supplementary Table S5). 

### 3.4. SNPs in the Vitamin D-Responsive Genes and CRC Risk

We also examined the associations between 434 SNPs in the genes responsive to vitamin D, including the genes in the TGFβ and IGF signaling pathways, inflammation, oxidative stress, cell cycle, and 19 SNPs located in the *VDR* binding sites as previously published [19] (Supplementary Table S6). Twenty-five SNPs were significantly associated with CRC risk at *P* < 0.01. However, after BH correction, none of the associations (except for *SMAD3* rs7180244; *SMAD7* rs11874392, rs12953717 and rs4939827) remained statistically significant (Supplementary Table S7). Interestingly, three SNPs (rs3197999, rs3802842, rs762421) in previously identified *VDR* binding sites were associated with CRC risk. The results did not differ by tumor location (Supplementary Tables S6 and S7) or sex (Supplementary Table S8). 

### 3.5. Vitamin D Genes/Pathways and CRC Risk

At the pathway level, the *VDR* binding sites and TGFβ signaling pathway were statistically significantly associated with CRC risk (*P* < 0.04; Table 4). For colon cancer, in addition to the *VDR* binding sites (*P* = 0.008) and TGFβ signaling pathway (*P* = 0.0001), an association with cell cycle pathway was observed (*P* = 0.03). The TGFβ (*P* = 0.0001) and IGF (*P* = 0.007) signaling pathways, but not the *VDR* binding sites (*P* = 0.256), were statistically significantly associated with rectal cancer risk. 

At the gene level, several genes (*CHAF1A, SMARCE1, SMAD7, SMAD3, BMP2*, and *C-MYC* region) were associated with CRC risk at unadjusted *P* < 0.05. However, all of them except *SMAD7* (*P_BH_* = 0.008) and *SMAD3* (*P_BH_* = 0.008) were not statistically significant after BH correction. The *SMAD7, SMAD3, BMP2*, and *C-MYC* regions were associated with colon cancer; however, after BH correction, only *SMAD7* (*P_BH_* = 0.04) and *SMAD3* (*P_BH_* = 0.009) remained statistically significant. In addition to *SMAD7* (*P* = 0.0005) and *SMAD3* (*P* = 0.0003), several other genes or genetic regions (*CYP2R1, CHAF1A, CREBBP, IL10*, SNPs identified in genome-wide association studies (GWAS) to be associated with IGF levels, *IGFBP2/IGFBP5, IGFBP3*, and *C-MYC* region) were associated with rectal cancer. However, after BH correction, only *SMAD7* (*P* = 0.02) and *SMAD3* (*P* = 0.02) remained statistically significant.

### 3.6. 25. (OH)D-Gene and 25(OH)D Pathway Interactions and CRC Risk

At the pathway level, the *VDR* complex and its transcriptional co-regulators and co-activators demonstrated a potential interaction with 25(OH)D concentrations in the association with CRC risk (*P* = 0.04; Table 4). Within this pathway, the interaction *P*-values of <0.05 were observed for *ARID1A, CARM1, CHAF1A*, and *SMARCA2*, but none were statistically significant after BH correction. Similar associations were observed for colon cancer, but not rectal cancer (*P* for interaction for the *VDR* complex and its transcriptional co-regulators and co-activators were 0.105 and 0.727, respectively).

At the gene level, the interaction *P*-values of <0.05 were observed for *CYP27B1* and *GC* (vitamin D metabolism) and *IL10* (inflammation) for CRC and colon cancer. Also, the interaction between 25(OH)D and *IGFBP2/IGFBP5* was statistically significant for colon cancer. For rectal cancer, the interaction *P*-values of <0.05 were observed for *CYP24A1* (vitamin D metabolism) and *BMP2* (TGF signaling). None of the gene-25(OH)D interactions were statistically significant after BH correction.

Next, we assessed the associations between 25(OH)D (per 24.96 nmol/L) with CRC risk, stratified by genotypes of SNPs in the genes that were identified in the step above as potentially modifying the association of 25(OH)D with CRC risk (*CYP27B1*, *GC*, *ARID1A, CARM1, CHAF1A*, *SMARCA2*, and *IL10;* Supplementary Table S9). Sixteen SNPs in these genes with *P* for interaction <0.05 are presented in Table 5. None were statistically significant after BH correction. Several SNPs had a very low number of minor allele homozygotes, with no effect estimates presented in the table.

## 4. Discussion

In this large European prospective case-control study nested within the EPIC cohort, we investigated whether genetic variation in the genes and pathways related to vitamin D metabolism and vitamin D genomic effects is associated with CRC risk, and whether these associations are modified by 25(OH)D concentrations. 

We identified several genes related to vitamin D metabolism, the VDR complex, and VDR transcriptional activity associated with 25(OH)D concentrations, with an unadjusted *P* < 0.01 before BH correction among controls. We confirmed two genes related to vitamin D metabolism, *CYP27B1* and *GC*, and one in the *VDR*, which were identified in previous GWAS studies [28,29,30,31,32]. We also identified other genes in our study including 1) two genes that encode the transcription-related factors *HDAC9* and *NCOA7*, involved in vitamin D transcriptional activity and VDR complex formation, and 2) two genes that encode the vitamin D-related transporters *LRP2* and *CUBN* [33,34]. *LRP2*, commonly known as megalin, is responsible for the endocytosis of the 25(OH)D vitamin D binding protein complex [35]. *CUBN* is an important co-receptor in the megalin-mediated endocytic pathway and patients without functioning CUBN were found to have abnormal 25(OH)D metabolism [33].

The genes associated with circulating 25(OH)D concentrations were also associated with CRC risk at unadjusted *P* < 0.01 before BH correction. *HDAC9* is located in a region on chromosome 7p21 [36] in which chromosomal gains were observed in primary CRC [37]. Furthermore, HDAC9 has been observed via chromatin immunoprecipitation (ChIP) assay in human osteosarcoma tissues to suppress p53 transcription and, thereby, promote cell proliferation [38]. An association of *CUBN* with CRC was previously reported in a meta-analysis of six GWAS studies [39], while no studies have investigated a possible association of *LRP2* with CRC. *LRP2* is expressed in multiple epithelial cell lines, including colon [35,40] and is often co-expressed with *CUBN* [34]. Additionally, there are no previous GWAS regarding CRC and *NCOA7*, although Higginbotham et al., found statistically significant associations of *NCOA7* gene variants with reduced breast cancer risk across three different study cohorts [41]. The *NCOA7* SNPs identified in our study, however, differed from those identified by Higginbotham suggesting a possible novel CRC susceptibility locus.

Three *VDR* binding site SNPs were associated with CRC risk in our study population, but the associations were not statistically significant after BH correction. We a priori selected these *VDR* binding sites for genotyping based on the results of a previous study that used ChIP followed by DNA sequencing to identify 2776 *VDR* binding sites in lymphoblastoid cell lines treated with calcitriol, an active form of vitamin D [19]. The study found a statistically significant 4-fold increase in the enrichment of *VDR* binding sites located in genes associated with CRC, and a 3.5-fold increase in enrichment located in genes associated with Crohn’s disease [19]. Our findings suggest that genetic variation in these *VDR* binding sites, upregulated in response to treatment with vitamin D and relevant to colorectal carcinogenesis and inflammatory bowel diseases, may be associated with CRC risk.

TGFβ has an important role in the regulation of cell proliferation, differentiation, migration and apoptosis [42], and may be modulated by vitamin D [43]. *SMAD7* and *SMAD3*, in the TGFβ signaling pathway, were statistically significantly associated with CRC risk after BH correction for multiple testing. *SMAD7* SNPs were previously identified to be associated with CRC risk in several different populations [44,45,46] as well as in a meta-analysis of 2906 cases and 3416 controls from four previous GWAS studies [47]. SMAD7 is transcriptionally induced by cytokines from the TGFβ family and regulates the TGFβ signaling pathway via a negative feedback loop [42]; therefore, the overexpression of SMAD7 inhibits the pathway and its associated anti-neoplastic effects [42]. The active form of vitamin D was shown to inhibit SMAD7 in experimental models [48]. The role of *SMAD3* in the development of CRC is less understood and somatic tumor mutations in this gene have been observed in only 4.3% of CRC cases [49]. SMAD3 has been identified to interact with *VDR* and mediate a cross-talk between TGFβ and vitamin D signaling pathways [50]. An animal model found that *SMAD3* may also play an important role in the TGFβ response to inflammation and bacteria-induced colon carcinogenesis [51]. Inflammation is further associated with CRC risk in our study as indicated by the interaction between circulating 25(OH)D concentrations, which has anti-inflammatory properties [52,53], and genetic variation in the *IL10* gene encoding anti-inflammatory cytokine interleukin (IL)-10 involved in immune response to pathogens [54]. The induction of IL-10 is mediated by 1,25(OH)_2_D and is repressed with SMAD3 inhibition [48].

Chromosome *8q24* polymorphisms in the cell cycle pathway were previously identified to be strongly associated with CRC risk [47,55,56]. However, they were not statistically significant after BH correction. Although the *8q24* region is described as a gene desert, it is closely located to the region encoding *c-MYC* oncogene [57]. *C-MYC* controls processes related to cell growth regulation, metabolism and proliferation and is not only activated by numerous oncogenic pathways but also stimulates metabolic changes which can lead to malignant transformation [58]. Multiple studies have identified long-range physical interaction of the *8q24* region with *c-MYC* via enhancer elements and chromatin loops [57,59,60]. In an experimental study, 1,25(OH)_2_D and the *VDR* were shown to affect the c-*MYC/MXD1* pathway leading to inhibition of c-MYC protein expression [61]. Using *8q24* SNPs as a proxy, our results confirm an association between *c-MYC* and CRC risk, but do not indicate a potential modification by 25(OH)D despite a previously reported possible interaction for fatal prostate cancer risk [62]. 

The IGF signaling pathway plays a key role in cell growth [63]. In our study, IGF-related genetic variation was associated with rectal cancer risk at the pathway level as well as for several individual genes before BH correction. Contrary to our results, IGF genetic variants [64,65] in addition to high circulating IGF peptides [66] have been previously associated more strongly with increased colon versus rectal cancer risk. 

The strengths of our study include its prospective design and high follow-up rate. The hypothesis-driven selection of pathways, genes, and SNPs, and relatively large samples size within a large cohort study allowed an extensive investigation of vitamin D–related and –responsive genetic variation and the effect modification by established biomarker of vitamin D status with CRC risk. We used the detailed data from EPIC to address potential confounding by body size and other factors; with our careful analyses suggesting no or little confounding. However, we cannot altogether discount the possibility of residual confounding nor changes in lifestyle habits between enrolment into the cohort and the eventual cancer diagnosis. Although our study was large, most interaction and stratified analyses had limited power, especially by sex and tumor location. Our power analyses (Supplementary Table S10) showed that we have sufficient power (80%) to detect the effect associations in the range of 1.17 to 1.27 for relatively common SNPs with MAF between 40 and 10%, respectively, using our full data set (n = 1419 matched case-control sets). In addition, most of our results were not statistically significant after BH correction for multiple testing. As to the selection of genes and pathways, we were limited by published literature on vitamin D at the time of genotyping, so we may have not included all vitamin D-responsive genes. Additional experimental studies are needed to understand the biological mechanisms of the identified associations.

## 5. Conclusions

This large and comprehensive study has confirmed genetic variations in several previously identified vitamin D-related pathways associated with CRC risk in European populations, and has suggested potential new pathways related to vitamin D genomic effects and colorectal carcinogenesis.

## Figures and Tables

**Table 1:** Selected baseline characteristics of incident colorectal cancer (CRC) cases and their matched controls, the European Prospective Investigation into Cancer and Nutrition (EPIC) study, 1992–2003.

Baseline Characteristic	Cases		Controls	
	n = 1420		n = 1419	
Women, N (%)	705	(49.6)	701	(49.4)
Mean age at blood collection, (SD) years	58.5	(7.3)	58.6	(7.3)
Mean years of follow-up, (SD) years	4.1	(2.3)	---	
Smoking status, N (%) ^a^				
Never	580	(40.8)	594	(41.9)
Former	476	(33.5)	460	(32.4)
Current	346	(24.4)	349	(24.6)
Physical activity, N (%)				
Inactive	202	(14.2)	183	(12.9)
Moderately inactive	402	(28.3)	367	(25.9)
Moderately active	583	(41.1)	612	(43.1)
Active	130	(9.2)	148	(10.4)
BMI, (SD) kg/m^2^	26.8	(4.2)	26.3	(3.8)
25-(OH)-vitamin D measurement, N (%)	1,176	(82.8)	764	(53.8)
25-(OH)-vitamin D, mean (SD) nmol/L ^b^	58.5	(25.6)	62.0	(25.4)
Country, N (%)				
France	28	(2.0)	29	(2.0)
Italy	202	(14.2)	198	(14.0)
Spain	146	(10.3)	141	(9.9)
United Kingdom	240	(16.9)	250	(17.6)
The Netherlands	153	(10.8)	158	(11.1)
Greece	46	(3.2)	48	(3.4)
Germany	179	(12.6)	169	(11.9)
Sweden	88	(6.2)	86	(6.1)
Denmark	338	(23.8)	340	(24.0)

^a^ Percent missing is not shown. Therefore the total percentages do not add up to 100%. ^b^ Season standardized using the sine-curve method [25].

**Table 2:** Top 10 single-nucleotide polymorphisms (SNPs) in the genes related to vitamin D metabolism and transcriptional activity associated with season-adjusted 25(OH)D concentrations among controls only, the EPIC study, 1992–2003 ^a^.

Gene ^b^	SNP	N	25(OH)D, β (95% CI)	*P*	*P_BH_* ^c^
*VDR*	rs2239182	742	−3.82 (−6.15, −1.49)	*0.001*	*0.949*
*LRP2*	rs2673170	747	−4.43 (−7.27, −1.58)	*0.002*	*0.949*
*NCOA7*	rs579477	758	−3.57 (−6.07, −1.07)	*0.005*	*0.949*
*GC*	rs1352844	747	5.26 (1.63, 8.88)	*0.005*	*0.949*
*GC*	rs188812	752	5.38 (1.57, 9.20)	*0.006*	*0.949*
*GC*	rs2298849	757	4.26 (1.11, 7.41)	*0.008*	*0.949*
*CUBN*	rs4525114	750	7.11 (1.94, 12.29)	*0.007*	*0.949*
*CYP27B1*	rs4646536	751	3.65 (0.93, 6.37)	*0.009*	*0.949*
*CYP27B1*	rs10877013	764	3.42 (0.73, 6.12)	*0.013*	*0.974*
*HDAC9*	rs212669	753	−8.12 (−14.34, −1.90)	*0.011*	*0.974*

^a^ Adjusted for age at blood collection, sex, and center. ^b^ Genes related to vitamin D metabolism and transcriptional activity. ^c^ *P* after Benjamini–Hochberg (BH) multiple testing correction.

**Table 3:** Associations of SNPs with CRC risk overall and by tumor location (colon vs. rectum), the EPIC study, 1992–2003.

Gene/SNP	Genotype	Colorectal Cancer					Colon Cancer				Rectal Cancer			
		Cases	Controls	OR (95% CI) ^a^	*P*	*P_BH_*	Cases	OR (95% CI) ^a^	*P*	*P_BH_*	Cases	OR (95% CI) ^a^	*P*	*P_BH_* ^b^
*CUBN*														
rs12243895	GG	702	767	1.00 (ref)	0.009	0.569	435	1.00 (ref)	0.006	0.509	267	1.00 (ref)	0.261	0.969
	GA	551	513	1.18 (1.00, 1.38)			354	1.20 (1.00, 1.44)			197	1.12 (0.90, 1.40)		
	AA	140	104	1.48 (1.12, 1.96)			94	1.59 (1.17, 2.16)			46	1.34 (0.91, 1.97)		
	Additive	1393	1384	1.20 (1.07, 1.35)	0.002	0.274	883	1.24 (1.08, 1.41)	0.002	0.254	510	1.14 (0.97, 1.34)	0.106	0.824
	Dominant	1393	1384	1.22 (1.05, 1.43)	0.009	0.561	883	1.27 (1.07, 1.50)	0.007	0.571	510	1.16 (0.94, 1.43)	0.169	0.896
	Recessive	1393	1384	1.38 (1.05, 1.80)	0.020	0.898	883	1.46 (1.08, 1.97)	0.013	0.827	510	1.27 (0.88, 1.85)	0.204	0.939
rs1801224	AA	601	669	1.00 (ref)	0.015	0.677	359	1.00 (ref)	0.004	0.473	242	1.00 (ref)	0.517	0.998
	AC	614	582	1.18 (1.00, 1.38)			399	1.26 (1.05, 1.51)			215	1.04 (0.84, 1.30)		
	CC	180	144	1.40 (1.09, 1.80)			120	1.52 (1.15, 2.01)			60	1.22 (0.87, 1.73)		
	Additive	1395	1395	1.18 (1.06, 1.32)	0.004	0.374	878	1.24 (1.09, 1.41)	0.001	0.169	517	1.08 (0.93, 1.27)	0.308	0.926
	Dominant	1395	1395	1.22 (1.05, 1.42)	0.010	0.561	878	1.31 (1.10, 1.56)	0.002	0.377	517	1.07 (0.87, 1.32)	0.494	0.965
	Recessive	1395	1395	1.29 (1.02, 1.63)	0.035	0.898	878	1.35 (1.04, 1.76)	0.026	0.875	517	1.20 (0.86, 1.67)	0.276	0.986
rs7096079	CC	275	338	1.00 (ref)	0.023	0.677	159	1.00 (ref)	0.007	0.509	116	1.00 (ref)	0.587	0.998
	CA	654	620	1.31 (1.08, 1.60)			417	1.43 (1.14, 1.80)			237	1.13 (0.87, 1.48)		
	AA	301	305	1.22 (0.97, 1.53)			194	1.36 (1.04, 1.77)			107	1.03 (0.75, 1.40)		
	Additive	1230	1263	1.10 (0.99, 1.24)	0.084	0.894	770	1.16 (1.02, 1.32)	0.025	0.712	460	1.02 (0.87, 1.18)	0.841	0.996
	Dominant	1230	1263	1.28 (1.07, 1.54)	0.008	0.561	770	1.41 (1.13, 1.75)	0.002	0.377	460	1.10 (0.86, 1.41)	0.461	0.965
	Recessive	1230	1263	1.02 (0.85, 1.22)	0.861	1.000	770	1.06 (0.86, 1.31)	0.578	0.991	460	0.95 (0.73, 1.22)	0.668	0.997
*VDR*														
rs886441	AA	885	926	1.00 (ref)	0.024	0.677	563	1.00 (ref)	0.028	0.729	322	1.00 (ref)	0.179	0.963
	AG	444	404	1.16 (0.98, 1.36)			273	1.12 (0.93, 1.35)			171	1.21 (0.97, 1.52)		
	GG	57	36	1.66 (1.08, 2.56)			40	1.83 (1.15, 2.93)			17	1.32 (0.73, 2.41)		
	Additive	1386	1366	1.20 (1.05, 1.38)	0.009	0.508	876	1.20 (1.03, 1.40)	0.020	0.670	510	1.19 (0.99, 1.44)	0.067	0.690
	Dominant	1386	1366	1.20 (1.02, 1.40)	0.027	0.693	876	1.18 (0.98, 1.41)	0.078	0.896	510	1.22 (0.99, 1.52)	0.067	0.750
	Recessive	1386	1366	1.59 (1.04, 2.43)	0.034	0.898	876	1.77 (1.11, 2.81)	0.016	0.827	510	1.24 (0.68, 2.25)	0.481	0.997
*NCOA2*														
rs10087049	AA	393	472	1.00 (ref)	0.007	0.569	240	1.00 (ref)	0.003	0.448	153	1.00 (ref)	0.180	0.963
	AG	724	665	1.32 (1.11, 1.56)			464	1.40 (1.15, 1.71)			260	1.17 (0.92, 1.48)		
	GG	173	182	1.14 (0.89, 1.46)			120	1.30 (0.98, 1.72)			53	0.88 (0.61, 1.26)		
	Additive	1290	1319	1.12 (1.00, 1.26)	0.054	0.848	824	1.19 (1.04, 1.36)	0.010	0.596	466	1.00 (0.85, 1.17)	0.959	0.996
	Dominant	1290	1319	1.28 (1.08, 1.51)	0.004	0.469	824	1.38 (1.14, 1.67)	0.001	0.297	466	1.11 (0.88, 1.39)	0.385	0.963
*NCOA7*														
rs10223441	CC	648	709	1.00 (ref)	0.007	0.569	399	1.00 (ref)	0.009	0.531	249	1.00 (ref)	0.153	0.939
	CG	640	561	1.25 (1.07, 1.47)			413	1.30 (1.09, 1.55)			227	1.17 (0.95, 1.45)		
	GG	128	149	0.93 (0.72, 1.21)			84	0.98 (0.72, 1.31)			44	0.85 (0.59, 1.23)		
	Additive	1416	1419	1.06 (0.95, 1.19)	0.277	0.921	896	1.09 (0.96, 1.24)	0.183	0.920	520	1.01 (0.87, 1.18)	0.882	0.996
	Dominant	1416	1419	1.19 (1.02, 1.38)	0.025	0.669	896	1.23 (1.04, 1.46)	0.017	0.685	520	1.10 (0.90, 1.35)	0.338	0.944
	Recessive	1416	1419	0.84 (0.65, 1.08)	0.169	0.948	896	0.86 (0.65, 1.14)	0.295	0.991	520	0.79 (0.55, 1.13)	0.202	0.939
rs17292488	GG	594	639	1.00 (ref)	0.004	0.569	375	1.00 (ref)	0.019	0.659	219	1.00 (ref)	0.031	0.882
	GA	657	575	1.23 (1.05, 1.44)			416	1.21 (1.01, 1.46)			241	1.26 (1.01, 1.57)		
	AA	148	185	0.86 (0.67, 1.10)			97	0.86 (0.65, 1.13)			51	0.85 (0.60, 1.21)		
	Additive	1399	1399	1.01 (0.90, 1.13)	0.841	0.998	888	1.00 (0.89, 1.14)	0.942	0.994	511	1.02 (0.88, 1.19)	0.802	0.996
	Dominant	1399	1399	1.14 (0.98, 1.33)	0.087	0.876	888	1.13 (0.95, 1.34)	0.174	0.944	511	1.16 (0.94, 1.43)	0.155	0.896
	Recessive	1399	1399	0.77 (0.61, 0.97)	0.028	0.898	888	0.77 (0.59, 1.01)	0.059	0.973	511	0.76 (0.54, 1.06)	0.102	0.939
*NCOR2*														
rs10846670	AA	288	360	1.00 (ref)	0.021	0.677	169	1.00 (ref)	0.007	0.509	119	1.00 (ref)	0.676	0.998
	AG	688	669	1.29 (1.07, 1.56)			441	1.39 (1.12, 1.74)			247	1.12 (0.86, 1.44)		
	GG	274	265	1.30 (1.03, 1.64)			177	1.42 (1.09, 1.86)			97	1.12 (0.81, 1.53)		
	Additive	1250	1294	1.15 (1.02, 1.29)	0.020	0.663	787	1.20 (1.05, 1.37)	0.007	0.564	463	1.06 (0.91, 1.24)	0.461	0.927
	Dominant	1250	1294	1.29 (1.08, 1.55)	0.005	0.528	787	1.40 (1.13, 1.73)	0.002	0.377	463	1.12 (0.87, 1.43)	0.377	0.962
	Recessive	1250	1294	1.09 (0.90, 1.32)	0.356	0.987	787	1.13 (0.91, 1.41)	0.256	0.991	463	1.04 (0.80, 1.36)	0.777	0.997
rs906304	GG	1032	1082	1.00 (ref)	0.010	0.569	666	1.00 (ref)	0.005	0.496	366	1.00 (ref)	0.025	0.827
	GA	359	298	1.26 (1.06, 1.51)			220	1.20 (0.98, 1.47)			139	1.37 (1.08, 1.74)		
	AA	20	32	0.66 (0.38, 1.17)			6	0.30 (0.13, 0.74)			14	1.36 (0.71, 2.62)		
	Additive	1411	1412	1.12 (0.96, 1.31)	0.141	0.894	892	1.02 (0.85, 1.22)	0.822	0.990	519	1.30 (1.07, 1.59)	0.010	0.514
	Dominant	1411	1412	1.21 (1.02, 1.43)	0.032	0.693	892	1.12 (0.92, 1.36)	0.268	0.954	519	1.37 (1.09, 1.73)	0.007	0.416
	Recessive	1411	1412	0.63 (0.35, 1.11)	0.106	0.931	892	0.29 (0.12, 0.70)	0.006	0.714	519	1.26 (0.66, 2.40)	0.490	0.997
*CHAF1A*														
rs243352	CC	410	369	1.00 (ref)	0.014	0.677	250	1.00 (ref)	0.240	0.977	160	1.00 (ref)	0.003	0.453
	CA	695	673	0.93 (0.78, 1.11)			438	0.97 (0.79, 1.19)			257	0.85 (0.67, 1.08)		
	AA	285	346	0.74 (0.60, 0.91)			190	0.82 (0.65, 1.05)			95	0.60 (0.44, 0.80)		
	Additive	1390	1388	0.86 (0.78, 0.96)	0.006	0.417	878	0.91 (0.81, 1.03)	0.128	0.920	512	0.78 (0.67, 0.90)	0.001	0.132
	Dominant	1390	1388	0.86 (0.73, 1.02)	0.087	0.876	878	0.92 (0.76, 1.11)	0.392	0.956	512	0.76 (0.61, 0.95)	0.018	0.629
	Recessive	1390	1388	0.77 (0.65, 0.92)	0.005	0.648	878	0.84 (0.69, 1.03)	0.097	0.981	512	0.66 (0.51, 0.86)	0.002	0.729
rs9352	AA	461	417	1.00 (ref)	0.023	0.677	277	1.00 (ref)	0.315	0.995	184	1.00 (ref)	0.003	0.453
	AG	648	681	0.86 (0.72, 1.02)			413	0.92 (0.76, 1.13)			235	0.75 (0.60, 0.95)		
	GG	254	307	0.74 (0.60, 0.92)			166	0.83 (0.65, 1.06)			88	0.61 (0.46, 0.83)		
	Additive	1363	1405	0.86 (0.78, 0.96)	0.006	0.417	856	0.91 (0.81, 1.03)	0.131	0.920	507	0.78 (0.67, 0.90)	0.001	0.132
	Dominant	1363	1405	0.82 (0.70, 0.97)	0.018	0.597	856	0.89 (0.74, 1.08)	0.238	0.954	507	0.71 (0.57, 0.88)	0.002	0.243
	Recessive	1363	1405	0.81 (0.68, 0.98)	0.032	0.898	856	0.87 (0.70, 1.07)	0.192	0.991	507	0.73 (0.56, 0.95)	0.018	0.939
*HDAC9*														
rs2520361	AA	881	841	1.00 (ref)	0.021	0.677	556	1.00 (ref)	0.161	0.960	325	1.00 (ref)	0.033	0.882
	AG	385	395	0.92 (0.78, 1.09)			240	0.93 (0.76, 1.13)			145	0.91 (0.72, 1.15)		
	GG	50	79	0.60 (0.41, 0.87)			36	0.68 (0.45, 1.02)			14	0.46 (0.26, 0.83)		
	Additive	1316	1315	0.85 (0.75, 0.97)	0.018	0.641	832	0.88 (0.75, 1.02)	0.086	0.920	484	0.81 (0.67, 0.98)	0.027	0.664
	Dominant	1316	1315	0.87 (0.74, 1.02)	0.089	0.876	832	0.89 (0.74, 1.07)	0.197	0.946	484	0.84 (0.67, 1.05)	0.122	0.853
	Recessive	1316	1315	0.61 (0.43, 0.88)	0.009	0.898	832	0.69 (0.46, 1.04)	0.079	0.973	484	0.48 (0.27, 0.85)	0.013	0.939
rs4141042	AA	1028	1072	1.00 (ref)	0.007	0.569	645	1.00 (ref)	0.006	0.509	383	1.00 (ref)	0.146	0.921
	AG	366	304	1.26 (1.06, 1.50)			238	1.30 (1.07, 1.58)			128	1.23 (0.97, 1.57)		
	GG	18	31	0.60 (0.33, 1.09)			10	0.54 (0.26, 1.12)			8	0.71 (0.32, 1.56)		
	Additive	1412	1407	1.11 (0.95, 1.30)	0.176	0.916	893	1.13 (0.95, 1.35)	0.166	0.920	519	1.10 (0.90, 1.36)	0.346	0.926
	Dominant	1412	1407	1.20 (1.01, 1.42)	0.039	0.752	893	1.23 (1.01, 1.49)	0.035	0.806	519	1.18 (0.93, 1.49)	0.164	0.896
	Recessive	1412	1407	0.57 (0.32, 1.03)	0.062	0.916	893	0.51 (0.25, 1.05)	0.067	0.973	519	0.67 (0.30, 1.49)	0.328	0.986
*SMARCC1*														
rs3755637	GG	661	605	1.00 (ref)	0.015	0.677	412	1.00 (ref)	0.026	0.729	249	1.00 (ref)	0.073	0.882
	GA	520	601	0.79 (0.67, 0.93)			322	0.78 (0.64, 0.93)			198	0.81 (0.65, 1.02)		
	AA	132	141	0.86 (0.66, 1.12)			90	0.95 (0.70, 1.27)			42	0.70 (0.48, 1.03)		
	Additive	1313	1347	0.87 (0.78, 0.98)	0.023	0.712	824	0.90 (0.79, 1.03)	0.111	0.920	489	0.83 (0.70, 0.97)	0.023	0.650
	Dominant	1313	1347	0.80 (0.69, 0.93)	0.005	0.517	824	0.81 (0.68, 0.96)	0.017	0.685	489	0.79 (0.64, 0.98)	0.030	0.679
	Recessive	1313	1347	0.96 (0.75, 1.24)	0.758	1.000	824	1.07 (0.80, 1.41)	0.659	0.991	489	0.78 (0.54, 1.12)	0.174	0.939
*TOP2B*														
rs1001647	AA	948	884	1.00 (ref)	0.022	0.677	612	1.00 (ref)	0.011	0.531	336	1.00 (ref)	0.460	0.993
	AG	353	415	0.79 (0.66, 0.93)			220	0.74 (0.61, 0.90)			133	0.87 (0.68, 1.10)		
	GG	57	55	0.94 (0.64, 1.39)			37	0.91 (0.59, 1.41)			20	1.06 (0.61, 1.81)		
	Additive	1358	1354	0.86 (0.75, 0.99)	0.032	0.792	869	0.83 (0.71, 0.97)	0.017	0.650	489	0.93 (0.77, 1.13)	0.454	0.926
	Dominant	1358	1354	0.80 (0.68, 0.95)	0.009	0.561	869	0.76 (0.63, 0.92)	0.004	0.442	489	0.89 (0.71, 1.11)	0.298	0.943
	Recessive	1358	1354	1.02 (0.70, 1.49)	0.922	1.000	869	1.00 (0.65, 1.55)	0.982	0.999	489	1.11 (0.65, 1.90)	0.702	0.997

^a^ Unconditional logistic regression adjusted for age at blood collection, sex, and study center. ^b^ *P* of false discovery rate (BH; Benjamini–Hochberg) method.

**Table 4:** P-values of pathway- and gene-level associations with CRC risk overall and by tumor location (colon vs. rectal) and of interactions with 25(OH)D concentrations (per 24.96 nmol/L), the EPIC study, 1992–2003.

Pathway/Gene	No. of SNPs	No. of SNPs Retained After Pruning	Colorectal Cancer				Colon Cancer				Rectal Cancer			
			Gene or Pathway Only		Gene- or Pathway-25(OH)D Interaction		Gene or Pathway Only		Gene- or Pathway-25(OH)D Interaction		Gene or Pathway Only		Gene- or Pathway-25(OH)D Interaction	
			*P*	*P_BH_* ^a^	*P*	*P_BH_*	*P*	*P_BH_*	*P*	*P_BH_*	*P*	*P_BH_*	*P*	*P_BH_*
**Vitamin D metabolism**	**276**	245	0.580		0.159		0.550		0.160		0.418		0.116	
Identified in GWAS of 25(OH)D ^b^	2	2	0.235	0.759	0.867	0.999	0.167	0.657	0.923	0.990	0.561	0.944	0.499	0.991
CUBN	116	106	0.173	0.741	0.764	0.999	0.130	0.657	0.896	0.990	0.083	0.470	0.490	0.991
CYP24A1	25	23	0.443	0.777	0.358	0.999	0.083	0.647	0.666	0.990	0.622	0.944	**0.007**	0.595
CYP27A1	5	5	0.500	0.777	0.299	0.999	0.488	0.819	0.086	0.522	0.968	0.968	0.256	0.991
CYP27B1	6	5	0.448	0.777	**0.037**	0.446	0.585	0.829	**0.041**	0.448	0.514	0.944	0.154	0.991
CYP2R1	12	9	0.115	0.741	0.811	0.999	0.368	0.815	0.921	0.990	**0.044**	0.459	0.727	0.991
CYP3A4	7	5	0.241	0.759	0.730	0.999	0.461	0.815	0.533	0.990	0.262	0.747	0.392	0.991
DHCR7	12	6	0.997	0.997	0.549	0.999	0.800	0.911	0.614	0.990	0.434	0.944	0.716	0.991
GC	24	20	0.484	0.777	**0.018**	0.406	0.912	0.954	**0.026**	0.442	0.241	0.747	0.316	0.991
LRP2	67	64	0.804	0.926	0.377	0.999	0.508	0.819	0.677	0.990	0.904	0.967	0.487	0.991
**Mineral homeostasis**	58	40	0.834		0.313		0.912		0.431		0.537		0.782	
CASR	31	23	0.580	0.784	0.736	0.999	0.536	0.819	0.957	0.990	0.643	0.944	0.565	0.991
PTH	6	5	0.931	0.982	0.671	0.999	0.773	0.911	0.739	0.990	0.539	0.944	0.736	0.991
CALB1	2	2	0.489	0.777	0.741	0.999	0.400	0.815	0.847	0.990	0.882	0.967	0.819	0.991
TRPV5	9	7	0.657	0.846	0.054	0.456	0.920	0.954	0.081	0.522	0.337	0.818	0.225	0.991
TRPV6	10	3	0.263	0.777	0.880	0.999	0.520	0.819	0.954	0.990	0.112	0.595	0.713	0.991
**VDR complex/Transcriptional Co-regulators and Co-activators**	538	490	0.634		**0.041**		0.874		0.105		0.180		0.727	
ACTL6A	3	3	0.239	0.759	0.395	0.999	0.262	0.815	0.497	0.990	0.506	0.944	0.613	0.991
ARID1A	8	7	0.408	0.777	**0.032**	0.446	0.306	0.815	**0.048**	0.448	0.133	0.628	0.068	0.924
BAZ1B	14	9	0.478	0.777	0.955	0.999	0.360	0.815	0.867	0.990	0.385	0.909	0.935	0.993
CARM1	4	4	0.641	0.839	**0.006**	0.406	0.831	0.929	**0.022**	0.442	0.290	0.747	0.120	0.991
CHAF1A	5	4	**0.035**	0.511	**0.013**	0.406	0.307	0.815	**0.047**	0.448	**0.007**	0.187	0.098	0.926
CREBBP	15	12	0.388	0.777	0.285	0.999	0.800	0.911	0.215	0.865	**0.011**	0.187	0.793	0.991
EP300	6	5	0.771	0.926	0.791	0.999	0.434	0.815	0.835	0.990	0.917	0.967	0.342	0.991
HDAC9	149	141	0.559	0.784	0.873	0.999	0.524	0.819	0.578	0.990	0.553	0.944	0.970	0.993
MED1	5	5	0.507	0.777	0.561	0.999	0.719	0.899	0.694	0.990	0.131	0.628	0.617	0.991
NCOA1	18	14	0.581	0.784	0.065	0.504	0.804	0.911	0.201	0.854	0.239	0.747	0.355	0.991
NCOA2	19	16	0.542	0.781	0.579	0.999	0.145	0.657	0.456	0.990	0.938	0.967	0.505	0.991
NCOA3	11	9	0.067	0.636	0.051	0.456	0.056	0.647	0.069	0.489	0.522	0.944	0.350	0.991
NCOA7	31	31	0.518	0.777	0.142	0.755	0.646	0.872	0.056	0.448	0.223	0.747	0.482	0.991
NCOR1	7	3	0.312	0.777	0.802	0.999	0.377	0.815	0.759	0.990	0.577	0.944	0.489	0.991
PCAF/KAT2B	31	31	0.801	0.926	0.891	0.999	0.470	0.815	0.865	0.990	0.938	0.967	0.797	0.991
PRMT1	4	4	0.346	0.777	0.351	0.999	0.407	0.815	0.184	0.823	0.618	0.944	0.624	0.991
RXRA	30	27	0.683	0.866	0.763	0.999	0.091	0.647	0.732	0.990	0.662	0.944	0.726	0.991
RXRB	7	3	0.824	0.926	0.074	0.526	0.445	0.815	0.058	0.448	0.617	0.944	0.356	0.991
RXRG	24	24	0.853	0.929	0.716	0.999	0.558	0.819	0.923	0.990	0.875	0.967	0.087	0.924
SMARCA2	1	1	0.506	0.777	**0.019**	0.406	0.381	0.815	**0.012**	0.442	0.944	0.967	0.307	0.991
SMARCA4	12	9	0.474	0.777	0.794	0.999	0.559	0.819	0.975	0.990	0.557	0.944	0.923	0.993
SMARCC1	4	4	0.103	0.741	0.893	0.999	0.344	0.815	0.824	0.990	0.082	0.470	0.164	0.991
SMARCD1	3	3	0.312	0.777	0.703	0.999	0.356	0.815	0.550	0.990	0.519	0.944	0.998	0.998
SMARCE1	4	4	**0.048**	0.582	0.197	0.881	0.191	0.706	0.390	0.990	0.083	0.470	0.059	0.924
SNW1	10	10	0.615	0.816	0.863	0.999	0.740	0.911	0.947	0.990	0.813	0.967	0.807	0.991
SUPT16H	7	6	0.809	0.926	0.990	0.999	0.696	0.899	0.960	0.990	0.663	0.944	0.841	0.991
TOP2B	6	5	0.192	0.741	0.965	0.999	0.097	0.647	0.327	0.990	0.281	0.747	0.400	0.991
NCOR2	62	61	0.701	0.877	0.960	0.999	0.770	0.911	0.609	0.990	0.459	0.944	0.935	0.993
VDR	30	28	0.270	0.777	0.652	0.999	0.372	0.815	0.707	0.990	0.477	0.944	0.706	0.991
TSC2	8	7	0.135	0.741	0.885	0.999	0.354	0.815	0.811	0.990	0.284	0.747	0.761	0.991
**TGF-beta signaling**	110	98	**0.0001**		0.616		**0.0001**		0.729		**0.0001**		0.342	
RHPN2	25	23	0.452	0.777	0.722	0.999	0.144	0.657	0.871	0.990	0.956	0.967	0.701	0.991
SMAD7	23	18	**0.0001**	**0.008**	0.927	0.999	**0.001**	**0.043**	0.947	0.990	**0.0005**	**0.021**	0.627	0.991
SMAD3	39	38	**0.0002**	**0.008**	0.367	0.999	**0.0001**	**0.009**	0.514	0.990	**0.0003**	**0.021**	0.085	0.924
BMP2	5	5	**0.016**	0.346	0.081	0.527	**0.014**	0.298	0.224	0.865	0.072	0.470	**0.049**	0.924
BMP4	1	1	0.201	0.742	0.431	0.999	0.334	0.815	0.615	0.990	0.261	0.747	0.276	0.991
TGFB1	11	8	0.159	0.741	0.293	0.999	0.469	0.815	0.112	0.595	0.068	0.470	0.306	0.991
TGFBR1	4	3	0.950	0.982	0.741	0.999	0.306	0.815	0.838	0.990	0.446	0.944	0.851	0.991
SCG5/GREM1	2	2	0.141	0.741	0.412	0.999	0.170	0.657	0.371	0.990	0.323	0.808	0.656	0.991
**Inflammation**	133	97	0.888		0.479		0.620		0.156		0.200		0.784	
ALOX5	22	11	0.839	0.926	0.264	0.999	0.801	0.911	0.593	0.990	0.782	0.967	0.165	0.991
IL10	13	8	0.462	0.777	**0.030**	0.446	0.854	0.931	**0.006**	0.442	**0.009**	0.187	0.419	0.991
IL10R	9	7	0.734	0.904	0.552	0.999	0.913	0.954	0.751	0.990	0.663	0.944	0.591	0.991
IL2/IL21	6	5	0.441	0.777	0.933	0.999	0.538	0.819	0.978	0.990	0.666	0.944	0.607	0.991
IL6	13	10	0.983	0.995	0.807	0.999	0.982	0.988	0.557	0.990	0.696	0.967	0.843	0.991
IL12B	18	17	0.128	0.741	0.870	0.999	0.079	0.647	0.796	0.990	0.791	0.967	0.761	0.991
IFNG	7	4	0.300	0.777	0.686	0.999	0.453	0.815	0.608	0.990	0.287	0.747	0.497	0.991
TNF	4	4	0.959	0.982	0.218	0.926	0.988	0.988	0.375	0.990	0.790	0.967	0.076	0.924
NFKB1	22	15	0.816	0.926	0.644	0.999	0.873	0.939	0.736	0.990	0.856	0.967	0.794	0.991
IL12A	1	1	0.523	0.777	0.168	0.795	0.452	0.815	0.159	0.770	0.814	0.967	0.706	0.991
IL18	4	4	0.348	0.777	0.514	0.999	0.613	0.840	0.766	0.990	0.182	0.737	0.290	0.991
IL1A/IL1B	11	8	0.951	0.982	0.124	0.701	0.846	0.931	0.054	0.448	0.761	0.967	0.711	0.991
IL8	1	1	0.153	0.741	0.978	0.999	0.060	0.647	0.593	0.990	0.811	0.967	0.669	0.991
RELA (p65)	2	2	0.921	0.982	0.835	0.999	0.938	0.961	0.962	0.990	0.922	0.967	0.690	0.991
**Oxidative Stress**	51	37	0.726		0.471		0.598		0.460		0.913		0.747	
GSR	9	7	0.530	0.777	0.116	0.701	0.712	0.899	0.110	0.595	0.656	0.944	0.376	0.991
GPx2	15	8	0.834	0.926	0.772	0.999	0.713	0.899	0.748	0.990	0.412	0.944	0.915	0.993
TXNRD1 (TR1)	17	14	0.232	0.759	0.859	0.999	0.168	0.657	0.875	0.990	0.757	0.967	0.946	0.993
SOD2	10	8	0.530	0.777	0.374	0.999	0.546	0.819	0.599	0.990	0.896	0.967	0.281	0.991
**Insulin growth factor (IGF) signaling**	61	52	0.105		0.320		0.550		0.135		**0.007**		0.346	
Associated with IGF levels in GWAS ^c^	4	4	0.414	0.777	0.999	0.999	0.579	0.829	0.901	0.990	**0.036**	0.437	0.879	0.993
IGF1	17	15	0.131	0.741	0.452	0.999	0.113	0.657	0.668	0.990	0.225	0.747	0.655	0.991
IGF2BP2	3	1	0.303	0.777	0.923	0.999	0.596	0.830	0.580	0.990	0.156	0.698	0.658	0.991
IGFBP2/IGFBP5	24	21	0.187	0.741	0.052	0.456	0.692	0.899	**0.018**	0.442	**0.029**	0.411	0.607	0.991
IGFBP3	13	11	0.057	0.601	0.882	0.999	0.273	0.815	0.997	0.997	**0.050**	0.459	0.058	0.924
**Cell Cycle**	60	56	0.120		0.852		**0.030**		0.845		0.482		0.916	
KRAS	13	13	0.511	0.777	0.498	0.999	0.262	0.815	0.436	0.990	0.182	0.737	0.925	0.993
FOS (c-fos)	10	9	0.389	0.777	0.425	0.999	0.230	0.815	0.518	0.990	0.764	0.967	0.574	0.991
JUN	7	7	0.569	0.784	0.407	0.999	0.411	0.815	0.163	0.770	0.917	0.967	0.811	0.991
C-MYC region ^d^	13	13	**0.010**	0.295	0.843	0.999	**0.051**	0.647	0.857	0.990	**0.054**	0.459	0.959	0.993
CCND1	4	3	0.177	0.741	0.893	0.999	0.155	0.657	0.565	0.990	0.549	0.944	0.763	0.991
BCL2A1	2	2	0.118	0.741	0.587	0.999	0.083	0.647	0.574	0.990	0.250	0.747	0.915	0.993
BAX	6	5	0.308	0.777	0.892	0.999	0.155	0.657	0.748	0.990	0.922	0.967	0.988	0.998
CDKN1A	5	4	0.521	0.777	0.168	0.795	0.099	0.647	0.459	0.990	0.867	0.967	0.216	0.991
**VDR binding sites**	19	18	**0.036**		0.530		**0.008**		0.410		0.256		0.798	
VDR binding sites ^e^	19	18	**0.036**	0.511	0.530	0.999	**0.008**	0.227	0.410	0.990	0.256	0.747	0.798	0.991

^a^*P* of false discovery rate (BH; Benjamini–Hochberg or BH) method. ^b^ rs10485165 and rs10507577 (Bejamin et al. 2007) [27]. ^c^ rs1245541, rs4234798, rs700752, and rs780094. ^d^ Chromosome 8q24 region. ^e^ SNPs located in the *VDR* binding sites relating to colorectal cancer and Crohn’s disease risk as previously published (Ramagopalan et al. 2010) [19].

**Table 5:** Associations of season-adjusted 25(OH)D concentrations (per 24.96 nmol/L) with CRC risk by genotypes, the EPIC study, 1992–2003.

Gene/SNP	Major Allele Homozygotes		Heterozygotes		Minor Allele Homozygotes		*P*_interaction_^b^
	Cases/Controls	OR (95%CI) ^a^	Cases/Controls	OR (95%CI) ^a^	Cases/Controls	OR (95%CI) ^a^	
**Vitamin D metabolism**							
CYP27B1 rs10877013	557/383	1.00 (0.86,1.17)	510/311	0.84 (0.73,0.97)	108/70	0.61 (0.40,0.93)	*0.024*
CYP27B1 rs4646536	551/381	1.00 (0.86,1.17)	485/300	0.85 (0.74,0.99)	110/70	0.62 (0.41,0.95)	*0.034*
GC rs1352846	500/319	0.96 (0.81,1.13)	406/286	0.79 (0.67,0.93)	104/68	0.58 (0.35,0.97)	*0.049*
GC rs16846876	530/331	1.05 (0.90,1.23)	477/337	0.75 (0.65,0.88)	144/81	0.88 (0.63,1.21)	*0.017*
GC rs2298850	584/384	1.00 (0.86,1.15)	445/293	0.80 (0.68,0.94)	86/61	0.56 (0.33,0.97)	*0.038*
GC rs3755967	569/359	0.99 (0.85,1.15)	436/297	0.78 (0.66,0.91)	101/64	0.66 (0.40,1.09)	*0.034*
GC rs842873	261/192	0.73 (0.58,0.91)	588/354	0.84 (0.73,0.97)	240/196	1.22 (0.97,1.52)	*0.002*
**VDR complex/Transcriptional Co-regulators and Co-activators**							
ARID1A rs11247596	720/474	0.96 (0.85,1.09)	400/256	0.76 (0.63,0.92)	53/34	0.42 (0.20,0.86)	*0.051*
ARID1A rs12737946	998/640	0.83 (0.74,0.93)	168/116	1.25 (0.95,1.66)	9/8	- ^c^	*0.016*
ARID1A rs12752833	998/641	0.83 (0.74,0.93)	165/114	1.22 (0.92,1.62)	9/8	-	*0.025*
CARM1 rs7254708	764/515	0.82 (0.73,0.93)	208/144	1.04 (0.81,1.32)	9/8	-	*0.0001*
CHAF1A rs243341	596/403	0.78 (0.68,0.90)	468/276	0.95 (0.80,1.13)	102/78	1.42 (0.97,2.07)	*0.020*
CHAF1A rs243365	607/434	0.80 (0.70,0.91)	408/257	1.02 (0.86,1.22)	50/44	1.64 (0.89,3.02)	*0.027*
SMARCA2 rs2296212	920/607	0.81 (0.72,0.91)	229/142	1.15 (0.89,1.48)	14/7	4.09 (0.29,58.01)	*0.035*
**Inflammation**							
IL10 rs3024509	1015/657	0.92 (0.83,1.03)	132/100	0.62 (0.45,0.85)	3/2	-	*0.024*
IL10 rs6686931	747/476	0.80 (0.70,0.91)	373/243	1.02 (0.86,1.20)	49/38	1.42 (0.59,3.39)	*0.029*

^a^ Adjusted for age at blood collection, sex, and center. ^b^ Interactions between SNPs and 25(OH)D stratifying by categories of 25(OH)D concentrations and assuming a log-additive model for genetic markers. ^c^ Not estimatable due to small sample size.

## Supplementary Materials

The following are available online at https://www.mdpi.com/2072-6643/11/8/1954/s1, Table S1: Characteristics of SNPs used in the study, Table S2: Vitamin D metabolism and transcriptional activity-related SNPs associated (unadjusted *P*-value < 0.05) with season-adjusted 25(OH)D concentrations among controls, the EPIC study, 1992–2003, Table S3A: Associations between SNPs in the genes involved in the vitamin D metabolism, mineral homeostasis/endocrine regulation of 1,25(OH)2D synthesis, and vitamin D transcriptional activity with season-adjusted 25(OH)D concentrations among controls, Table S3B: Associations between SNPs in the genes involved in the vitamin D metabolism, mineral homeostasis/endocrine regulation of 1,25(OH)2D synthesis, and vitamin D transcriptional activity with season-adjusted 25(OH)D concentrations among cases and controls combined, Table S4: Associations of SNPs with CRC risk overall and by tumor location (colon vs. rectum) using unconditional logistic regression with adjustment for age at recruitment, study center and sex, the EPIC study, 1992–2003, Table S5 Associations of SNPs with CRC risk overall and by tumor location (colon vs. rectum) among men and women using unconditional logistic regression with adjustment for age at recruitment, study center and sex, the EPIC study, 1992–2003, Table S6: Associations of SNPs in vitamin D-responsive genes with CRC risk overall and by tumor location (colon vs. rectum) using unconditional logistic regression with adjustment for age at recruitment, study center and sex, the EPIC study, 1992-2003, Table S7: Statistically significant associations (unadjusted *p* < 0.01) of SNPs in vitamin D-responsive genes with CRC risk overall and by tumor location (colon vs. rectum) using unconditional logistic regression with adjustment for age at recruitment, study center and sex, the EPIC study, 1992–2003, Table S8: Associations of SNPs in vitamin D-responsive genes with CRC risk overall and by tumor location (colon vs. rectum) among men and women using unconditional logistic regression with adjustment for age at recruitment, study center and sex, the EPIC study, 1992–2003, Table S9: Associations of season-adjusted 25(OH)D concentrations (per 24.96 nmol/L) with CRC risk by genotypes, the EPIC study, 1992–2003, Table S10: SNP-Only Minimal Detectable Effect Associations by Minor Allele Frequency for 80% Power and N = 1419 matched cases and controls, the EPIC study, 1992–2003.

## Abbreviations

CRC—colorectal cancer; MR—Mendelian randomization; RCT—randomized control trial; VDR—vitamin D receptor; 1,25(OH)2D—1,25-dihydroxyvitamin D; 25(OH)D—25-hydroxyvitamin D; SNP—single nucleotide polymorphism; EPIC—European Prospective Investigation into Cancer and Nutrition; IGF—insulin growth factor; TGFβ—tumor growth factor β; MAF—minor allele frequency; tagSNPs—tagging SNPs; BMI—body mass index; ARTP—Adaptive Rank Truncated Product; BH—Benjamini–Hochberg; ChIP—chromatin immunoprecipitation.
